# The *AtFLC*-*AtFT* Pathway Is Involved in the Early Flowering Promoted by Loss of *AtHO1* Function in *Arabidopsis*

**DOI:** 10.3390/cimb48060587

**Published:** 2026-06-02

**Authors:** Quan Gu, Wenyang Zhang, Ziping Chen, Na Li, Shuwen Xu

**Affiliations:** 1 Anhui Promotion Center for Technology Achievements Transfer, Anhui Academy of Science and Technology, Hefei 230031, China; 2 School of Biology, Food and Environment, Hefei University, Hefei 230601, China; 3 School of Biological and Food Engineering, Suzhou University, Suzhou 234000, China

**Keywords:** heme oxygenase, flowering, *AtFLC*, *AtFT*, Arabidopsis

## Abstract

Although previous studies have indicated that heme oxygenase 1 (HO1/HY1) regulates the flowering time via the photoperiod pathway, the specific mechanism is still elusive. Here, we found that the Arabidopsis *hy1-100* mutant displayed early flowering, and the characteristic expression patterns of several master genes involved in the autonomous pathway were altered. Notably, the transcript levels of *FLOWERING LOCUS C* (*AtFLC*) gene declined developmentally in both wild-type and *hy1-100* mutant, with a more pronounced fold reduction observed in the mutant. Genetic evidence further underlined that *hy1-100/FLCOE* plants partially reversed the early flowering phenomenon of *hy1-100* mutant, suggesting that *AtHO1* regulated flowering at least partially through the AtFLC-involved autonomous pathway, as supported by changes in FLOWERING LOCUS T (AtFT) and SUPPRESSOR OF OVEREXPRESSION OF CO1 (AtSOC1) transcripts. Further analysis of *hy1-100/ft* mutants revealed that *hy1-100/ft* and *ft* mutants displayed similar late flowering phenotypes, accompanied by downregulated APETALA1 (*AtAP1*) and *AtSOC1*, indicating that *AtFT* played a crucial role in *AtHO1*-regulated flowering. Two key conclusions are drawn: first, the loss of *AtHO1* function promotes early flowering in Arabidopsis, which was genetically linked to the autonomous pathway regulating AtFLC expression; second, *AtFT* was an essential downstream factor mediating AtHO1-regulated flowering.

## 1. Introduction

The transition from vegetative to reproductive development is a very critical phase in the life cycle of higher plants. It is coordinately affected by the regulatory network of multiple external and internal factors. In the model plant, Arabidopsis, there are at least five distinct genetic pathways controlling flowering time, such as photoperiod pathway, autonomous pathway, the vernalization pathway, gibberellin acid (GA) pathway, and endogenous (age) pathway [1,2]. These pathways ultimately regulate the floral integrator genes, *FLOWERING LOCUS T (FT)* and *SUPPRESSOR OF CONSTANS 1 (SOC1)* [3,4]. Then, they activate the expression of floral meristem identity genes, *APETALA1 (AP1)* and *LEAFY (LFY)*, to trigger the generation of flowers in plants [5,6]. The regulation of photoperiod flowering is largely dependent on the light signal and internal circadian clock. Multiple key components, such as *CRYPTOCHROME1 (CRY1)*, *CRY2*, *PHYTOCHROMEA (PHYA)*, *PHYB*, *CONSTANS (CO)*, *EARLY FLOWERING 3 (ELF3)*, *ELF4*, and *GIGANTEA (GI)*, have essential roles in the photoperiod pathway [7,8,9,10,11]. It has been demonstrated that *ELF3* regulates the biological activity of GI by mediating the E3 ubiquitin ligase COP1 [8,11]. ELF4 interacts with GI to prevent the association of GI with the promoter of *CO*, thereby controlling flowering [9,10,11]. As a zinc-finger transcriptional activator, *CO* activates the expression of *FT* and *SOC1* to promote flowering under long days [12]. *FT* is also regulated by *FLOWERING LOCUS C* (*FLC*; which encodes a MADS box protein) [13]. It has a central role in the timing of flowering via binding to *FT* and repressing *FT* expression in the autonomous/vernalization pathway [14]. In addition, *FLC* has five paralogs, namely *MADS AFFECTING FLOWERING 1* (*MAF1*, also named *FLOWERING LOCUS M* (*FLM*), or *AGAMOUS-LIKE 27* (*AGL27*)), *MAF2*, *MAF3*, *MAF4*, and *MAF5* [15,16,17]. They also have significant functions in controlling flowering under certain conditions.

In the autonomous pathway, multiple genes (e.g., *FCA*, *FVE*, *FLOWERING LOCUS D* (*FLD*), *LUMINIDEPENDENS* (*LD*), and *FY*) promote flowering by repressing the expression of FLC via chromatin or RNA modification [18,19,20,21]. For example, FLOWERING LOCUS D (FLD), a histone Lys-4 demethylase, associates with FLC chromatin and is responsible for demethylation of H3K4me2 in the *FLC* gene body [18]. FCA, an RNA-binding protein, requires FLD to promote flowering by suppressing *FLC* expression [20]. FVE, a homolog of human (Homo sapiens) retinoblastoma-associated protein, interacts with the histone deacetylase HDA6 and inhibits *FLC* expression by promoting the deacetylation of FLC chromatin [22]. Furthermore, FLD is associated with HDA6 deacetylase and FVE, and the FLD-FVE- HDA6 complex is required for controlling transport in plants [21].

Heme oxygenase (HO/HY; EC 1.14.99.3) catabolizes heme to biliverdin with the release of carbon monoxide and free iron [23]. It is necessary for phytochrome chromophore biosynthesis and retrograde signaling in plants [24]. AtHO1 (AtHY1), the most highly expressed and inducible HO in Arabidopsis, was involved in a wide array of cellular adaptation and developmental processes [25]. It was confirmed that PHOTOPERIOD SENSITIVITY5 (SE5), encoding heme oxygenase with high similarity to AtHO1, controlled flowering by affecting both *heading date1* (*Hd1*) and *early heading date1* (*Ehd1*) in the photoperiodic flowering pathway in rice [26,27,28]. Moreover, HY1 played a critical role in nitrogen-dependent signaling, thereby modulating clock function and flowering performance under long-day conditions [28].

In this study, we reported the function of *AtHO1* in *Arabidopsis* flowering. In addition to the changes in AtELF3 and AtELF4 expression, the expression of *AtFLD*, *AtFCA*, and *AtFVE* genes was positively regulated in the *hy1-100* mutant. Genetic evidence further showed that AtFLC acted as a downstream regulator of *AtHO1* to mediate the expression of *AtFT* and *AtSOC1*. Moreover, *AtFT* was a limiting factor in the early flowering phenomenon of the *hy1-100* mutant. Therefore, *AtHO1* controlled flowering, at least partially via the AtFLC-AtFT-involved autonomous pathway, and a signaling step through which the autonomous pathway and photoperiodic flowering pathway interact might exist in plants.

## 2. Materials and Methods

### 2.1. Plant Materials and Growth Conditions

The Arabidopsis (Arabidopsis thaliana) *hy1-100* (CS236; Col-0) mutant was a mutant with a point mutation and was gifted from Profs. Wenbiao Shen and Yanjie Xie, Department of Plant Science, Nanjing Agricultural University. *FLCOE* (TPT_5.10140.1D; CS2102348; Col-0) and *ft* (SAIL_295_F04; CS866592; Col-0) mutants were obtained from AraShare (https://www.arashare.cn/index accessed on 7 May 2026).

The *hy1-100/FLCOE line* was obtained by crossing *hy1-100* with *FLCOE*, and the *hy1-100/ft* double mutant line was obtained by crossing *hy1-100* with *ft*. To isolate the *hy1-100/FLCOE* double mutant, we pollinated *hy1-100* pistils with pollen from *FLCOE*. The F_2_ progeny were first screened on hygromycin-containing medium, and the expression levels of *AtHO1* and *AtFLC* were then quantified to confirm the genotype. To isolate the *hy1-100/ft* double mutant, we pollinated *hy1-100* pistils with pollen from *ft*. To confirm the genotypes of the *hy1-100/ft* double mutant, we performed PCR-based genotyping using two primer sets, LP+RP and LB1+RP (Supplementary Table S1). The wild-type (WT) showed only the LP+RP product, and both the *ft* and *hy1-100/ft* mutants lacked the LP+RP band but exhibited a clear LB1+RP product, indicating homozygous T-DNA insertion in the *FT* gene (Supplementary Figure S1).

Seeds were surface-sterilized and washed with sterile water for 5 min three times, then cultured on solid half-strength Murashige and Skoog (MS) medium (pH 5.8). After for 4 d, seedlings were transplanted to soil in a growth chamber with a 16 h light/8 h dark (22 °C) regime at a light intensity of 150 mmol photons m^−2^ s^−1^ irradiation.

### 2.2. Flowering Time Phenotype Measurement

The flowering time was determined by counting the number of rosette leaves at the day floral buds became visible and the days at bolting, as described in [29]. At least 30 plants were measured and averaged for statistical treatment.

### 2.3. Gene Expression Analysis

Total RNA was extracted from leaves using a TransZol Up Kit (TransGen Biotech, Beijing, China) according to the manufacturer’s instructions. RNA concentration and quality were checked using the NanoDrop 2000 (Thermo Fisher Scientific, Wilmington, DE, USA). Complementary DNAs were synthesized from 1 μg of total RNA using an oligo(dT) primer and All-in-One First-Strand cDNA Synthesis SuperMix for qPCR (One-Step gDNA Removal) (TransGen Biotech). By using gene-specific primers (Supplementary Table S1, [30,31]), real-time quantitative reverse transcription (RT)-PCR was conducted using a CFX96 Real-Time System (Bio-Rad) with TransStart Green qPCR SuperMix (TransGen Biotech) according to the manufacturer’s instructions. Real-time RT-PCR was performed with a program (initial denaturation at 94 °C for 30 s, 94 °C for 5 s, and 60 °C for 30 s, 40 cycles). Melting curve analysis (65–95 °C, 0.5 °C increments) was performed to confirm primer specificity. The expression levels of genes were normalized to the *Tubulin2* gene. Relative gene expressions were calculated using the 2^−ΔΔCt^ method [32].

### 2.4. Statistical Analysis

Statistical analysis was performed using SPSS 18.0 software. Values were means ± SEM of three independent experiments, with at least three replicates for each. For statistical analysis, data were analyzed by Student’s *t*-test (* *p* ≤ 0.05, ** *p* ≤ 0.01, *** *p* ≤ 0.001, **** *p* ≤ 0.0001), or one-way ANOVA, followed by Tukey’s multiple range test, and *p* < 0.05 was considered statistically significant.

## 3. Results

### 3.1. Loss of AtHO1 Function Accelerated Flowering

**Figure 1:**
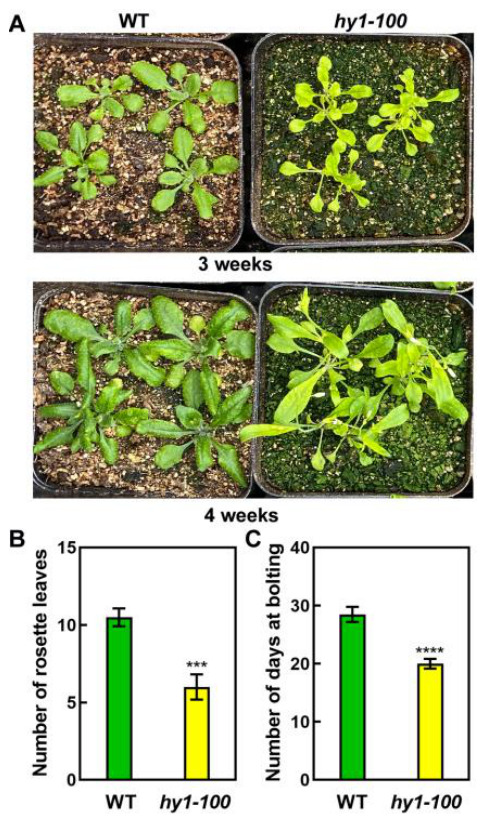
The early flowering phenotype of *hy1-100* mutant. (**A**) Three- and four-week-old wild-type (WT, Col) and *hy1-100* mutant plants grown under the same conditions (16 h light/8 h dark). (**B**,**C**) The number of rosette leaves on the day floral buds became visible and the days at bolting. Standard deviations (n ≥ 30) are shown. Significant differences between WT and *hy1-100* are indicated: *** *p* ≤ 0.001, **** *p* ≤ 0.0001 (Student’s *t*-test).

To identify the role of the *AtHO1* gene in regulating flowering in *Arabidopsis*, the early flowering phenotype of the *hy1-100* mutant (Col-0) was measured by the rosette leaf number at the flowering time and the days at bolting (Figure 1A). Under normal growth conditions, the *hy1-100* mutant flowered with an average of six leaves compared with 10 leaves of the wild-type Col-0 under the same conditions (16 h light/8 h dark) (Figure 1B). In addition, the *hy1-100* mutant plants bolted nearly 20 d after sowing, compared with 28 d for wild-type Col-0 plants (Figure 1C). Similar phenotype changes were also observed in the *hy1* mutant (Ler) and compared with wild-type Ler (Supplemental Figure S2). Therefore, the gene *AtHO1* was related to flowering time in *Arabidopsis*.

### 3.2. Expression of Genes Involved in the Autonomous Pathway Was Regulated in hy1-100 Mutant

**Figure 2:**
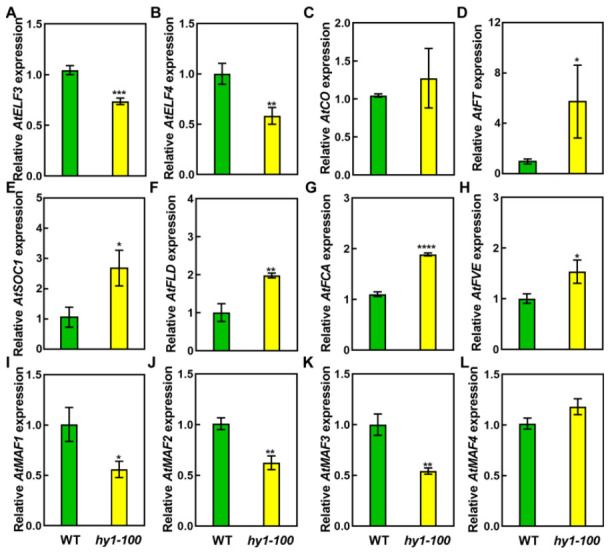
Expression analysis of flowering time genes in *hy1-100* mutant. (**A**–**L**) The relative expression levels of *AtELF3* (**A**), *AtELF* (**B**), *AtCO* (**C**), *AtFT* (**D**), *AtSOC1* (**E**), *AtFLD* (**F**), *AtFCA* (**G**), *AtFVE* (**H**), *AtMAF1* (**I**), *AtMAF2* (**J**), *AtMAF3* (**K**), and *AtMAF4* (**L**) genes were determined by real-time RT-PCR in leaves of 2-week-old wild-type (WT, Col) and *hy1-100* mutants under the same conditions (16 h light/8 h dark). Gene expression values are presented relative to the WT level, and were normalized using *Tubulin2* gene as internal control. Values are means ± SEM of results from three biological replicates. Significant differences between WT and *hy1-100* are indicated: * *p* ≤ 0.05, ** *p* ≤ 0.01, *** *p* ≤ 0.001, **** *p* ≤ 0.0001 (Student’s *t*-test).

To investigate the molecular mechanism underlying the early flowering phenotype of the *hy1-100* mutant, we compared the expression of several key genes involved in the photoperiod (*AtELF3*, *AtELF4*, and *AtCO*), as well as the *AtFT* and *AtSOC1* genes, between the *hy1-100* mutant and wild-type plants (Figure 2A–E). As expected, the expression of *AtELF3* and *AtELF4* genes was downregulated in the *hy1-100* mutant compared with wild-type plants (Figure 2A,B). There were no significant differences in *AtCO* expression (Figure 2C). Nevertheless, the two floral integrators, *AtFT* and *AtSOC1*, were accelerated significantly in the *hy1-100* mutant (Figure 2D,E).

**Figure 3:**
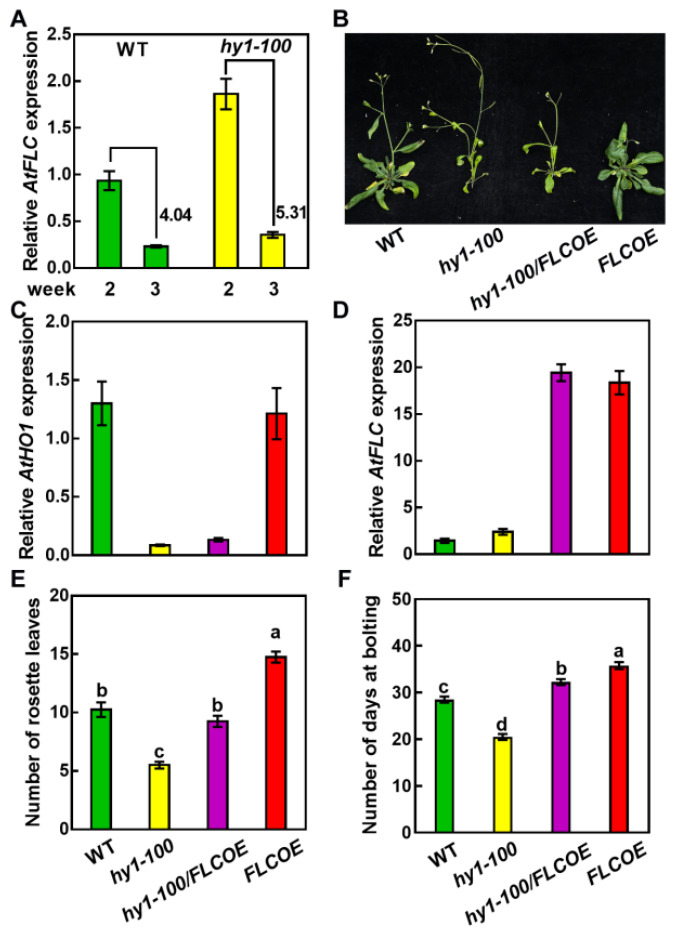
FLCOE transgenic plants can reverse the early flowering phenotype of the *hy1-100* mutant. (**A**) *AtFLC* is downregulated in both wild-type (WT, Col) and *hy1-100* mutants under the condition (16 h light/8 h dark) for 3 weeks. (**B**) WT, *hy1-100*, *hy1-100/FLCOE*, and *FLCOE* plants grown for 5 weeks. (**C**,**D**) The relative expression levels of *AtHO1* (**C**) and *AtFLC* (**D**) genes were determined by real-time RT-PCR in leaves of above 2-week-old genotypes. (**E**,**F**) The number of rosette leaves on the day floral buds became visible and the days at bolting. Standard deviations (n ≥ 30) are shown. Gene expression values are presented relative to the WT level, and were normalized using the *Tubulin2* gene as an internal control. Values are means ± SEM of results from three biological replicates. Bars with different letters denote significant differences (one-way ANOVA followed by Tukey’s multiple range test, *p* < 0.05).

Previous studies suggested that *AtFT* was known to be more dependent on the photoperiod pathway, while *AtSOC1* was more dependent on the autonomous pathway through *AtFLC* [33,34]. To determine the role of the autonomous pathway in the early flowering process of the *hy1-100* mutant, we compared the expression of several key genes involved in the autonomous (*AtFLD*, *AtFCA*, and *AtFVE*) pathway, and those genes universally related to *AtFLC*, *AtMAF1*, *AtMAF2*, *AtMAF3*, and *AtMAF4* genes (Figure 2F–L and Figure 3A). *AtFLD*, *AtFCA*, and *AtFVE* genes were upregulated substantially in the *hy1-100* mutant compared with wild-type plants (Figure 2F–H). Meanwhile, *AtMAF1*, *AtMAF2*, and *AtMAF3* genes exhibited the opposite tendencies (Figure 2I–K). The *AtMAF4* gene was not altered (Figure 2L). In addition, compared to wild-type plants, the *hy1-100* mutant exhibited consistently higher *AtFLC* expression at both 2 and 3 weeks, and was accompanied by a greater magnitude of developmental downregulation (Figure 3A). These results suggested that the autonomous pathway might be associated with the early flowering phenotype of the *hy1-100* mutant.

### 3.3. AtFLC Acted as a Downregulator of AtHO1 During Flowering

It was suggested that *AtFLC* acted as a rheostat that determined the capacity of flowering in Arabidopsis [35]. To further investigate the contribution of *AtFLC* in AtHO1-regulated flowering, the effect of an *AtFLC* overexpression line on the phenotype of the *hy1-100* mutant was determined (Figure 3B–F). Accordingly, we crossed the *FLCOE* line into the *hy1-100* mutant and identified the expression of *AtHO1* and *AtFLC* genes by real-time RT-PCR (Figure 3B–D). Then, we analyzed the flowering times of above genotype plants. The early flowering phenotype of the *hy1-100* mutant was reversed by the *FLCOE* line, and *hy1-100/FLCOE* flowered with a similar number of rosette leaves at flowering as the wild-type plants (Figure 3E,F). These results implied that *AtHO1* and *AtFLC* acted in the same pathway, and *AtHO1* acted upstream of *AtFLC* in the regulation of flowering.

### 3.4. Expression of AtFT and AtSOC1 Genes Was Downregulated by AtFLC in hy1-100 Mutant

**Figure 4:**
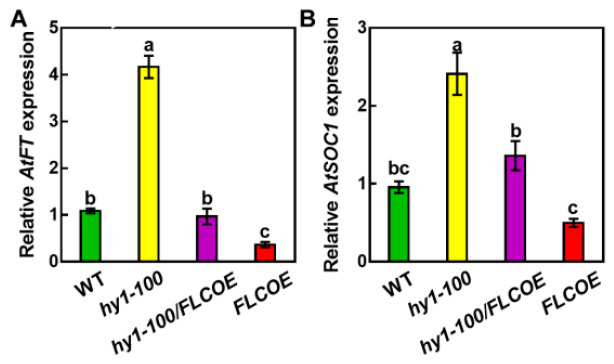
Expression of *AtFT* and *AtSOC1* is downregulated by *FLCOE* in *hy1-100* mutant. (**A**,**B**) The relative expression levels of *AtFT* (**A**) and *AtSOC1* (**B**) genes were determined by real-time RT-PCR in leaves of 2-week-old wild-type (WT, Col), *hy1-100*, *hy1-100/FLCOE*, and *FLCOE* plants under the condition (16 h light/8 h dark). Gene expression values are presented relative to the WT level, and were normalized using the *Tubulin2* gene as an internal control. Values are means ± SEM of results from three biological replicates. Bars with different letters denote significant differences (one-way ANOVA followed by Tukey’s multiple range test, *p* < 0.05).

To test whether the loss of *AtHO1* function promoted *AtFT* and *AtSOC1* expression via *AtFLC*, the expression of *AtFT* and *AtSOC1* in the *hy1-100/FLCOE* plants was analyzed. As shown in Figure 4, the transcripts of *AtFT* and *AtSOC1* decreased significantly in the *hy1-100/FLCOE* plants, and there were similar levels of *AtFT* and *AtSOC1* transcripts in both the *hy1-100/FLCOE* and wild-type plants. These results demonstrated that the early flowering phenotype of the *hy1-100* mutant plants was, at least partially, genetically related to *AtFLC* expression.

### 3.5. AtFT Acted as an Important Determinant in the Early Flowering Phenotype of hy1-100 Mutant

**Figure 5:**
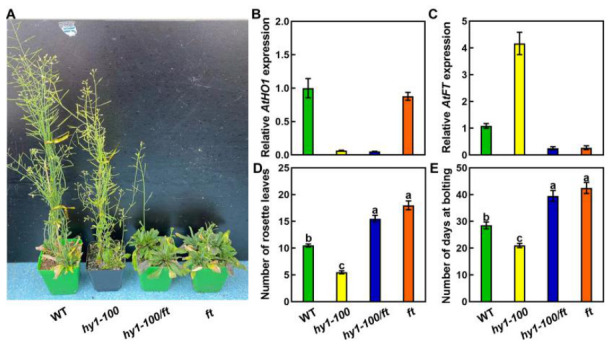
*ft* mutant can reverse the early flowering phenotype of the *hy1-100* mutant. (**A**) Wild-type (WT, Col), *hy1-100*, *hy1-100/ft*, and *ft* plants grown for 6 weeks. (**B**,**C**) The relative expression levels of *AtHO1* (**B**) and *AtFT* (**C**) genes were determined by real-time RT-PCR in leaves of 2-week-old genotypes. Gene expression values are presented relative to the WT level, and were normalized using the *Tubulin2* gene as an internal control. Values are means ± SEM of results from three biological replicates. (**D**,**E**) The number of rosette leaves on the day floral buds became visible and the days at bolting. Standard deviations (n ≥ 30) are shown. Bars with different letters denote significant differences (one-way ANOVA followed by Tukey’s multiple range test, *p* < 0.05).

Loss of *SE5* function, which encodes heme oxygenase with high similarity to *AtHO1*, upregulated the rice floral integrator Heading date3a (*Hd3a*), the ortholog gene of *AtFT* [26]. Then, we tested if *AtFT* is required for the early flowering phenotype of the *hy1-100* mutant plants. Accordingly, we crossed the *ft* mutant into the *hy1-100* mutant and identified the expression of *AtHO1* and *AtFT* genes by real-time RT-PCR (Figure 5A–C). Then, we analyzed the flowering times of the above genotype plants. The early flowering phenotype of the *hy1-100* mutant was reversed by the *ft* mutant, and the *hy1-100/ft* double mutant flowered with a similar number of rosette leaves at flowering and days at bolting as the *ft* mutant plants (Figure 5D,E).

**Figure 6:**
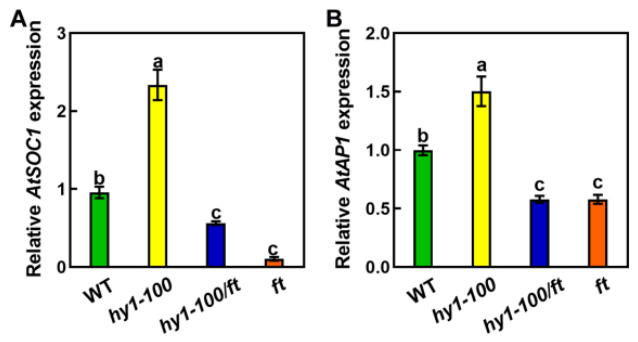
Expression of *AtSOC1* and *AtAP1* is downregulated in the *hy1-100/ft* mutant. (**A**,**B**) The relative expression levels of *AtSOC1* (**A**) and *AtAP1* (**B**) genes were determined by real-time RT-PCR in leaves of 2-week-old wild-type (WT, Col), *hy1-100*, *hy1-100/ft*, and *ft* plants under the condition (16 h light/8 h dark). Gene expression values are presented relative to the WT level, and were normalized using the *Tubulin2* gene as an internal control. Values are means ± SEM of results from three biological replicates. Bars with different letters denote significant differences (one-way ANOVA followed by Tukey’s multiple range test, *p* < 0.05).

Afterwards, the expression of *AtSOC1* and *AtAP1* was also analyzed. As shown in Figure 6, the transcripts of *AtSOC1* and *AtAP1* were decreased significantly in the *hy1-100/ft* double-mutant plants, and there were similar levels of *AtSOC1* and *AtAP1* transcripts in both the *hy1-100/ft* and *ft* mutant plants, showing that the ability of *AtHO1* deficiency to promote the expression of *AtSOC1* and *AtAP1* was dependent on the promotion of *AtFT* expression. Collectively, these findings provided genetic evidence that the early flowering phenotype of *hy1-100* mutant plants was genetically related to the promotion of *AtFT* expression.

## 4. Discussion

Previous studies revealed that heme oxygenase (HO/HY) converted the heme group into biliverdin IXα to synthesize the bilin chromophore used to assemble photochemically active phys in a number of plants [36,37]. Therefore, many studies focused on the role of the photoperiod pathway in HO1-regulated flowering [26,27,28,38,39]. For example, PHOTOPERIOD SENSITIVITY 5 (SE5), encoding a heme oxygenase, inhibited flowering by affecting both Hd1 and Ehd1 flowering pathways in rice [26,27,37]. Furthermore, loss of OsELF3 function suppressed early flowering in *se5* plants [38]. In this study, we observed that both *hy1-100* and *hy1* mutants showed an early flowering phenotype (Figure 1 and Supplemental Figure S2). We also found a decrease in *AtELF3* and *AtELF4* expression in the *hy1-100* mutant (Figure 2A,B). It was different from the role of OsELF3 and OsELF4, whereas *AtELF3* and *AtELF4* negatively regulated flowering in Arabidopsis [40,41]. These results suggested that the *AtELF3-* and *AtELF4*-regulated circadian clock might be involved in AtHO1-mediated flowering. Nevertheless, no obvious changes in *AtCO* expression were found in *hy1-100* mutant and wild-type plants (Figure 2C). Moreover, the relative expression level of its target, *AtFT,* was significantly increased in the *hy1-100* mutant compared with wild-type plants (Figure 2D). Hence, there might exist other pathways involved in AtHO1-regulated flowering.

### 4.1. AtHO1 Affected AtFLC Involved the Autonomous Pathway

It is known that *FT* expression is regulated by two central upstream regulators, CO and FLC [12,13]. Acting as a strong flowering repressor, *FLC* can repress the transcriptional activation of the floral integrator genes *FT* and *SOC1* in Arabidopsis [13]. The genes in the autonomous pathway, for example, *FLD*, *FCA*, and *FVE*, promote flowering by suppressing *FLC* [18,20,22]. Mutation in *FLD* resulted in hyperacetylation of histones in *FLC* chromatin, thus upregulating *FLC* expression and delaying flowering [18]. This present study further indicated that the expression levels of above autonomous pathway upstream genes were influenced in the *hy1-100* mutant (Figure 2F–H). Interestingly, the *hy1-100* mutant showed constitutively higher transcripts of the *AtFLC* gene than wild-type controls. Despite this elevation, the mutant still displayed a substantial reduction in *AtFLC* transcript abundance between the 2- and 3-week time points (Figure 3A). In addition, FLC paralogs (MAF1-MAF5) also have significant functions in controlling flowering [15,16,17]. In this study, we found that the transcripts of *AtMAF1*, *AtMAF2*, and *AtMAF3* genes in the *hy1-100* mutant were significantly lower than in wild-type plants, and *AtMAF4* expression was unchanged (Figure 2I–L). These results indicated that *AtHO1* was likely genetically linked to *AtFLC* expression. The typical autonomous pathway genes, known to regulate *AtFLC* via histone and RNA modifications, may mediate this putative relationship.

Further data obtained, working and comparing with wild-type, *hy1-100* mutant, *hy1-100/FLCOE*, and *FLCOE* plants, provided genetic evidence that *AtHO1*-regulated flowering was required for *AtFLC* expression, at least partially in Arabidopsis. This conclusion was supported by the results, showing that *AtFLC* inhibited the early flowering phenotype of the *hy1-100* mutant, and *hy1-100/FLCOE* flowered with a similar number of rosette leaves at flowering as wild-type plants (Figure 3B–F). Consistently, results shown in Figure 4 reveal that the transcripts of *AtFT* and *AtSOC1* in the *hy1-100* mutant were largely abolished by *FLCOE* plants, and were similar in both the *hy1-100/FLCOE* and wild-type plants. These results further deduced that the early flowering phenotype of the *hy1-100* mutant plants was genetically associated with the *AtFLC*-regulated autonomous pathway.

There are several floral-transition pathways that depend on the regulation of *FLC* expression to modulate flowering, directly or indirectly, for example, the vernalization pathway, gibberellin acid (GA) pathway, endogenous (age) pathway, BR pathway, and ABA pathway [1,2,42,43]. Among these, ABSCISIC ACID-INSENSITIVE 4 (*AtABI4*) negatively regulated flowering through directly promoting *AtFLC* transcription [43]. Evidence showed that *AtHO1* functioned negatively and acted upstream of *AtABI4* in drought signaling [44]. Therefore, the roles of *AtABI4* for *AtFLC* expression and the process of *AtHO1*-regulated flowering could not be easily ruled out, and should be investigated in a further study.

### 4.2. Loss of AtHO1 Function Led to Early Flowering Dependent on the AtFLC-AtFT-Mediated Pathway

The fact that *hy1-100* mutant plants flower earlier than wild-type plants was probably due to an additive effect of both a sharp decrease in *AtFLC* and increase in *AtFT* expression (Figure 2D, Figure 3A and Figure 4A). The early flowering phenotype of the hy1-100 mutant reverted when *AtFLC* was overexpressed, indicating that *AtFLC* acted downstream of *AtHO1* (Figure 3). Moreover, the transcripts of *AtFT* and *AtSOC1* in the *hy1-100* mutant were largely abolished by FLCOE plants. It has been confirmed that *AtFLC* encodes a MADS box protein that interacts directly in vivo with AtFT and AtSOC1 chromatin to repress their expression, hence regulating flowering [13,14]. It is possible that the flowering behavior was highly derived and dominated by the FT pathway in Arabidopsis [2,3]. Further, the *hy1-100/ft* double mutant showed a late flowering phenotype similar to the *ft* mutant plant, confirmed by the changes in both *AtSOC1* and *AtAP1* expression (Figure 5 and Figure 6). This led us to conclude that loss of *AtHO1* function was likely associated with early flowering, potentially through the AtFLC-AtFT pathway. It is worth pointing out that *AtFT* might be the crucial downstream component in this process.

## 5. Conclusions

**Figure 7:**
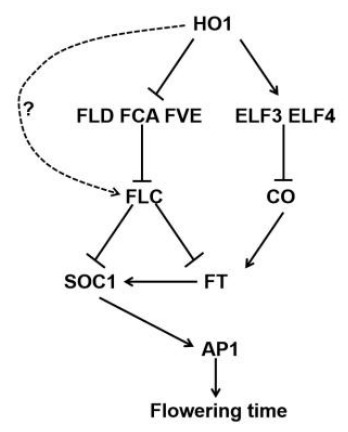
Diagram summarizing an AtHO1 working model to control flowering time in Arabidopsis. Lines with arrowheads indicate gene activation, and T bars indicate inhibition. The other proposed role of AtHO1 is indicated by dashed lines. HO1, heme oxygenase 1; FLD, FLOWERING LOCUS D; FCA, FLOWERING CONTROL LOCUS A; ELF3, EARLY FLOWERING 3; ELF4, EARLY FLOWERING 4; FLC, FLOWERING LOCUS C; CO, CONSTANS; FT, FLOWERING LOCUS T; SOC1, SUPPRESSOR OF OVEREXPRESSION OF CO1; AP1, APETALA1.

In summary, our genetic and molecular results indicated a genetic correlation connecting the AtFLC-AtFT-mediated autonomous pathway to the early flowering trait of the Arabidopsis *hy1-100* mutant (Figure 7). These findings enriched our understanding of pathway crosstalk in plant flowering control and the underlying complex regulatory networks. Our data supported a genetic association between *AtHO1* and the autonomous pathway, and it is likely that other signaling participants modulate the AtHO1-AtFT relationship. For example, the molecular mechanisms between *AtHO1* (an enzyme that can produce biliverdin and carbon dioxide) in chloroplasts and the nuclear autonomous pathway mechanism require further investigation. In particular, it is worth exploring whether there is an association between the activity of the AtHO1 enzyme and the transcription/epigenetic changes at the FLC locus in the future.

## Data Availability

The original contributions presented in this study are included in the article/supplementary material. Further inquiries can be directed to the corresponding author.

## Supplementary Materials

The following supporting information can be downloaded at: https://www.mdpi.com/article/10.3390/cimb48060587/s1.

## References

[1] Boss P.K., Bastow R.M., Mylne J.S., Dean C. (2004). Multiple pathways in the decision to flower: Enabling, promoting, and resetting. Plant Cell.

[2] Srikanth A., Schmid M. (2011). Regulation of flowering time: All roads lead to rome. Cell Mol. Life Sci..

[3] Corbesier L., Vincent C., Jang S., Fornara F., Fan Q., Searle I., Giakountis A., Farrona S., Gissot L., Turnbull C. (2007). FT protein movement contributes to long-distance signaling in floral induction of Arabidopsis. Science.

[4] Moon J., Suh S.S., Lee H., Choi K.R., Hong C.B., Paek N.C., Kim S.G., Lee I. (2003). The SOC1 MADS-box gene integrates vernalization and gibberellin signals for flowering in Arabidopsis. Plant J..

[5] Mandel M.A., Gustafson-Brown C., Savidge B., Yanofsky M.F. (1992). Molecular characterization of the Arabidopsis floral homeotic gene APETALA1. Nature.

[6] Liu C., Thong Z., Yu H. (2009). Coming into bloom: The specification of floral meristems. Development.

[7] Yanovsky M.J., Kay S.A. (2002). Molecular basis of seasonal time measurement in Arabidopsis. Nature.

[8] Yu J.W., Rubio V., Lee N.Y., Bai S., Lee S.Y., Kim S.S., Liu L., Zhang Y., Irigoyen M.L., Sullivan J.A. (2008). COP1 and ELF3 control circadian function and photoperiodic flowering by regulating GI stability. Mol. Cell.

[9] Kim Y., Lim J., Yeom M., Kim H., Kim J., Wang L., Kim W.Y., Somers D.E., Nam H.G. (2013). ELF4 regulates GIGANTEA chromatin access through subnuclear sequestration. Cell Rep..

[10] Kim Y., Yeom M., Kim H., Lim J., Koo H.J., Hwang D., Somers D., Nam H.G. (2012). GIGANTEA and EARLY FLOWERING 4 in Arabidopsis exhibit differential phase-specific genetic influences over a diurnal cycle. Mol. Plant.

[11] Zhao H., Xu D., Tian T., Kong F., Lin K., Gan S., Zhang H., Li G. (2021). Molecular and functional dissection of EARLY-FLOWERING 3 (ELF3) and ELF4 in Arabidopsis. Plant Sci..

[12] Samach A., Onouchi H., Gold S.E., Ditta G.S., Schwarz-Sommer Z., Yanofsky M.F., Coupland G. (2000). Distinct roles of CONSTANS target genes in reproductive development of Arabidopsis. Science.

[13] Hepworth S.R., Valverde F., Ravenscroft D., Mouradov A., Coupland G. (2002). Antagonistic regulation of flowering-time gene SOC1 by CONSTANS and FLC via separate promoter motifs. EMBO J..

[14] Helliwell C.A., Wood C.C., Robertson M., Peacock W.J., Dennis E.S. (2006). The Arabidopsis FLC protein interacts directly in vivo with SOC1 and FT chromatin and is part of a high-molecular-weight protein complex. Plant J..

[15] Ratcliffe O.J., Nadzan G.C., Reuber T.L., Riechmann J.L. (2001). Regulation of flowering in Arabidopsis by an FLC homologue. Plant Physiol..

[16] Ratcliffe O.J., Kumimoto R.W., Wong B.J., Riechmann J.L. (2003). Analysis of the Arabidopsis mads affecting flowering gene family: MAF2 prevents vernalization by short periods of cold. Plant Cell.

[17] Gu X., Le C., Wang Y., Li Z., Jiang D., Wang Y., He Y. (2013). Arabidopsis FLC clade members form flowering-repressor complexes coordinating responses to endogenous and environmental cues. Nat. Commun..

[18] Wu Z., Ietswaart R., Liu F., Yang H., Howard M., Dean C. (2016). Quantitative regulation of FLC via coordinated transcriptional initiation and elongation. Proc. Natl. Acad. Sci. USA.

[19] Wu Z., Fang X., Zhu D., Dean C. (2020). Autonomous pathway: FLOWERING LOCUS C repression through an antisense-mediated chromatin-silencing mechanism. Plant Physiol..

[20] Liu F., Quesada V., Crevillén P., Bäurle I., Swiezewski S., Dean C. (2007). The Arabidopsis RNA-binding protein FCA requires a lysine-specific demethylase 1 homolog to downregulate FLC. Mol. Cell.

[21] Yu C.W., Chang K.Y., Wu K. (2016). Genome-wide analysis of gene regulatory networks of the FVE-HDA6-FLD complex in Arabidopsis. Front. Plant Sci..

[22] Gu X., Jiang D., Yang W., Jacob Y., Michaels S.D., He Y. (2011). Arabidopsis homologs of retinoblastoma-associated protein 46/48 associate with a histone deacetylase to act redundantly in chromatin silencing. PLoS Genet..

[23] Davis S.J., Kurepa J., Vierstra R.D. (1999). The Arabidopsis thaliana HY1 locus, required for phytochrome-chromophore biosynthesis, encodes a protein related to heme oxygenases. Proc. Natl. Acad. Sci. USA.

[24] Muramoto T., Kohchi T., Yokota A., Hwang I., Goodman H.M. (1999). The Arabidopsis photomorphogenic mutant hy1 is deficient in phytochrome chromophore biosynthesis as a result of a mutation in a plastid heme oxygenase. Plant Cell.

[25] Mahawar L., Shekhawat G.S. (2018). Haem oxygenase: A functionally diverse enzyme of photosynthetic organisms and its role in phytochrome chromophore biosynthesis, cellular signalling and defence mechanisms. Plant Cell Environ..

[26] Andrés F., Galbraith D.W., Talón M., Domingo C. (2009). Analysis of PHOTOPERIOD SENSITIVITY5 sheds light on the role of phytochromes in photoperiodic flowering in rice. Plant Physiol..

[27] Rao Y., Xu N., Li S., Hu J., Jiao R., Hu P., Lin H., Lu C., Lin X., Dai Z. (2019). PE-1, encoding heme oxygenase 1, impacts heading date and chloroplast development in rice (*Oryza sativa* L.). J. Agric. Food Chem..

[28] Duan X., Wang G., Song Q., Liu J., Ding H., Che J., Xuan W. (2026). Long hypocotyl 1 mediates nitrogen regulation of flowering time and nitrogen use efficiency. Plant Sci..

[29] Mockler T., Yang H., Yu X., Parikh D., Cheng Y.C., Dolan S., Lin C. (2003). Regulation of photoperiodic flowering by Arabidopsis photoreceptors. Proc. Natl. Acad. Sci. USA.

[30] Wang B., Jin S.H., Hu H.Q., Sun Y.G., Wang Y.W., Han P., Hou B.K. (2012). UGT87A2, an Arabidopsis glycosyltransferase, regulates flowering time via FLOWERING LOCUS C. New Phytol..

[31] Berr A., Shafiq S., Pinon V., Dong A., Shen W.H. (2015). The trxG family histone methyltransferase SET DOMAIN GROUP 26 promotes flowering via a distinctive genetic pathway. Plant J..

[32] Livak K.J., Schmittgen T.D. (2001). Analysis of relative gene transcription data using real-time quantitative PCR and the 2^−△△CT^ method. Methods.

[33] Koornneef M., Alonso-Blanco C., Blankestijn-de Vries H., Hanhart C.J., Peters A.J. (1998). Genetic interactions among late-flowering mutants of Arabidopsis. Genetics.

[34] Moon J., Lee H., Kim M., Lee I. (2005). Analysis of flowering pathway integrators in Arabidopsis. Plant Cell Physiol..

[35] Simpson G.G. (2004). The autonomous pathway: Epigenetic and post-transcriptional gene regulation in the control of Arabidopsis flowering time. Curr. Opin. Plant Biol..

[36] Davis S.J., Bhoo S.H., Durski A.M., Walker J.M., Vierstra R.D. (2001). The heme-oxygenase family required for phytochrome chromophore biosynthesis is necessary for proper photomorphogenesis in higher plants. Plant Physiol..

[37] Emborg T.J., Walker J.M., Noh B., Vierstra R.D. (2006). Multiple heme oxygenase family members contribute to the biosynthesis of the phytochrome chromophore in Arabidopsis. Plant Physiol..

[38] Itoh H., Tanaka Y., Izawa T. (2019). Genetic relationship between phytochromes and OsELF3-1 reveals the mode of regulation for the suppression of phytochrome signaling in rice. Plant Cell Physiol..

[39] Izawa T., Oikawa T., Sugiyama N., Tanisaka T., Yano M., Shimamoto K. (2002). Phytochrome mediates the external light signal to repress FT orthologs in photoperiodic flowering of rice. Genes Dev..

[40] Doyle M.R., Davis S.J., Bastow R.M., McWatters H.G., Kozma-Bognar L., Nagy F., Millar A.J., Amasino R.M. (2002). The ELF4 gene controls circadian rhythms and flowering time in Arabidopsis thaliana. Nature.

[41] Zagotta M.T., Hicks K.A., Jacobs C.I., Young J.C., Hangarter R.P., Meeks-Wagner D.R. (1996). The Arabidopsis ELF3 gene regulates vegetative photomorphogenesis and the photoperiodic induction of flowering. Plant J..

[42] Li Z., Ou Y., Zhang Z., Li J., He Y. (2018). Brassinosteroid signaling recruits histone 3 lysine-27 demethylation activity to FLOWERING LOCUS C chromatin to inhibit the floral transition in Arabidopsis. Mol. Plant.

[43] Shu K., Chen Q., Wu Y., Liu R., Zhang H., Wang S., Tang S., Yang W., Xie Q. (2016). ABSCISIC ACID-INSENSITIVE 4 negatively regulates flowering through directly promoting Arabidopsis FLOWERING LOCUS C transcription. J. Exp. Bot..

[44] Xie Y., Mao Y., Duan X., Zhou H., Lai D., Zhang Y., Shen W. (2016). Arabidopsis HY1-modulated stomatal movement: An integrative hub is functionally associated with ABI4 in dehydration-induced ABA responsiveness. Plant Physiol..

