# Supplementary material for: The AtFLC-AtFT Pathway Is Involved in the Early Flowering Promoted by Loss of AtHO1 Function in Arabidopsis
Source: Curr Issues Mol Biol. 2026 Jun 2;48(6):587. doi: 10.3390/cimb48060587 (PMC13298054; doi:10.3390/cimb48060587)
Supplement: Supplementary file 1 [file cimb-48-00587-s001.zip › cimb-4334911-supplementary.pdf]

Supplementary Table S1 The sequences of primers for real-time RT-PCR in *Arabidopsis*.

Primer name	Locus number	Sequences (5’→3’)
AtHO1FP	AT2G26670	GAATCCCCAACTCTCAAG
AtHO1RP		TCATAGCCACAAACCTCA
AtFTFP	AT1G65480	AGGCCTTCTCAGGTTCAAAACAAGC
AtFTRP		TGCCAAAGGTTGTTCCAGTTGTAGC
AtFLCFP	AT5G10140	GCCAAGAAGACCGAACTCAT
AtFLCRP		TTTGTCCAGCAGGTGACATC
AtELF3FP	AT2G25930	CCATTGCCAATCAACAAAGAG
AtELF3RP		TGGTCAGTCTTCTCCGAGTCAC
AtELF4FP	AT2G40080	CGATGTGGGAGAATCTTGAC
AtELF4RP		TGTCGTTGACTTGTTGAATCAGTG
AtCOFP	AT5G15840	TCCCCCGTAGCTCGTCTGTGGTA
AtCORP		GCGTGCTCCGGCTGCTTTTT
AtSOC1FP	AT2G45660	ATAGGAACATGCTCAATCGAGGAGCTG
AtSOC1RP		TTTCTTGAAGAACAAGGTAACCCAATG
AtFVEFP	AT2G19520	CATCTGGGATTATGACAGGGTC
AtFVERP		TCCAAGGGTCTGAAGCATTC
AtFCAFP	AT4G16280	GCTCTTGTCGCAGCAAACTC
AtFCARP		GATCCAGCCCACTGTTGTTTAC
AtFLDFP		CTGACGCAGTGACTCGTGTTC
AtFLDRP	AT3G10390	GCTCCCACTGCAACATTAGAG
AtMAF1FP		GGAAAGAATACGTTGCTGGCAACA
AtMAF1RP	AT5G13240	CCGTTGATGATGGTGGCTAATTGA
AtMAF2FP	AT5G65050	GGCTCCGGAAAACTCTACAA
AtMAF2RP		TTCTGCAAGATCTAAGGCTTCA
AtMAF3FP		ACAGAACTAATGATGGAGGATATGAA

AtMAF3RP	AT5G65060	CTTCTTCCCCACCTGGCTA
AtMAF4FP	AT5G65075	GAGCAATGTCACCGGAAAGTAG
AtMAF4RP		CAGTCGTTGGTGATGGTGGTTA
AtAPFP	AT1G69120	CAGCAGCACCAAATCCAGC
AtAPRP		GAGCCTAGCCACTATTTATATG
AtTubulin2FP	AT5G05620	GAGCCTTACAACGCTACTCTGTCTGTC
AtTubulin2RP		ACACCAGACATAGTAGCAGAAATCAAG
LB1		GCCTTTTCAGAAATGGATAAATAGCCTT GCTTCC
ftLP	AT1G65480	ACTTTTCCATAATATGGCCGC
ftRP		GAACGTCTCCAACAACTCTGC

Supplementary Figures

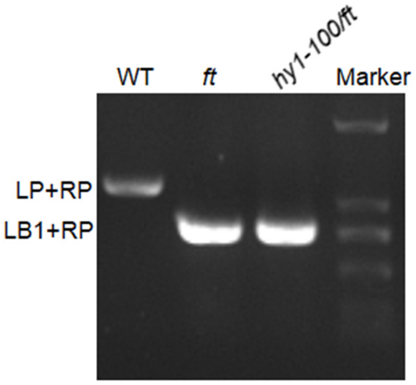

Supplementary Figure S1 Genotyping of the *ft* single mutant and *hy1-100/ft* double mutant by PCR-based analysis. PCR amplification of *FT* gene was performed by two primer pairs, gene-specific primers LP+RP were used to detect the wild-type allele, and the primers LB1+ RP were used to detect the T-DNA insertion allele. The wild-type (WT) control showed the LP+RP product, and both the *ft* mutant and *hy1-100/ft* double mutant exhibited a clear LB1+RP product and showed the homozygous T-DNA insertion in the *FT* gene. The Marker lane contains a DNA size ladder.

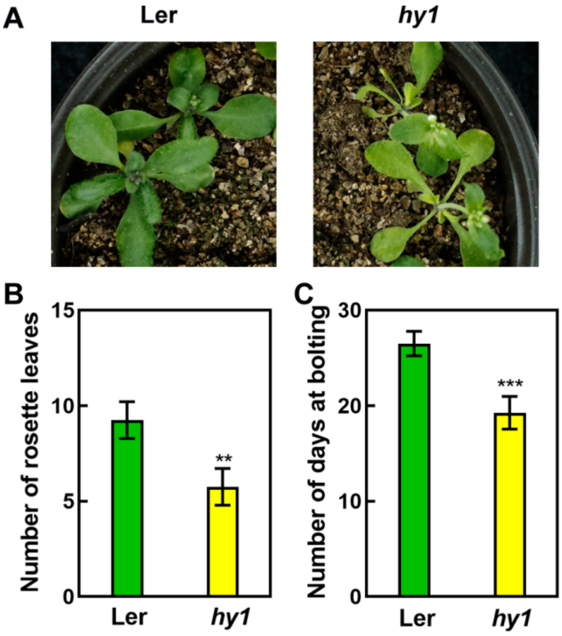

Supplementary Figure S2 The early-flowering phenotype of *hy1* mutant. (A) 4-week-old wild-type (WT, Ler) and *hy1* mutant plants grown under long days (LD;

16-h light/8-h dark). (B-C) The number of rosette leaves at the day floral buds became visible and the days at bolting. Standard deviations ($n \geqslant 30$) were shown. Significant differences between WT and *hy1* were indicated: $^{**}P \leqslant 0.01$, $^{***}P \leqslant 0.001$ (Student's t-test).
